# Biomechanical Loading Modulates Proinflammatory and Bone Resorptive Mediators in Bacterial-Stimulated PDL Cells

**DOI:** 10.1155/2014/425421

**Published:** 2014-05-25

**Authors:** Andressa Vilas Boas Nogueira, Marjan Nokhbehsaim, Sigrun Eick, Christoph Bourauel, Andreas Jäger, Søren Jepsen, Carlos Rossa, James Deschner, Joni Augusto Cirelli

**Affiliations:** ^1^Department of Diagnosis and Surgery, School of Dentistry at Araraquara, Univ Estadual Paulista (UNESP), 14801-903 Araraquara, SP, Brazil; ^2^Section of Experimental Dento-Maxillo-Facial Medicine, Center of Dento-Maxillo-Facial Medicine, University of Bonn, 53111 Bonn, Germany; ^3^Clinical Research Unit 208, Center of Dento-Maxillo-Facial Medicine, University of Bonn, 53111 Bonn, Germany; ^4^Department of Periodontology, Oral Microbiology, University of Bern, 3010 Bern, Switzerland; ^5^Oral Technology, Center of Dento-Maxillo-Facial Medicine, University of Bonn, 53111 Bonn, Germany; ^6^Department of Orthodontics, Center of Dento-Maxillo-Facial Medicine, University of Bonn, 53111 Bonn, Germany; ^7^Department of Periodontology, Operative and Preventive Dentistry, Center of Dento-Maxillo-Facial Medicine, University of Bonn, 53111 Bonn, Germany

## Abstract

The present study aimed to evaluate *in vitro* whether biomechanical loading modulates proinflammatory and bone remodeling mediators production by periodontal ligament (PDL) cells in the presence of bacterial challenge. Cells were seeded on BioFlex culture plates and exposed to *Fusobacterium nucleatum* ATCC 25586 and/or cyclic tensile strain (CTS) of low (CTSL) and high (CTSH) magnitudes for 1 and 3 days. Synthesis of cyclooxygenase-2 (COX2) and prostaglandin E2 (PGE2) was evaluated by ELISA. Gene expression and protein secretion of osteoprotegerin (OPG) and receptor activator of nuclear factor kappa-B ligand (RANKL) were evaluated by quantitative RT-PCR and ELISA, respectively. *F. nucleatum* increased the production of COX2 and PGE2, which was further increased by CTS. *F. nucleatum*-induced increase of PGE2 synthesis was significantly (*P* < 0.05) increased when CTSH was applied at 1 and 3 days. In addition, CTSH inhibited the *F. nucleatum*-induced upregulation of OPG at 1 and 3 days, thereby increasing the RANKL/OPG ratio. OPG and RANKL mRNA results correlated with the protein results. In summary, our findings provide original evidence that CTS can enhance bacterial-induced syntheses of molecules associated with inflammation and bone resorption by PDL cells. Therefore, biomechanical, such as orthodontic or occlusal, loading may enhance the bacterial-induced inflammation and destruction in periodontitis.

## 1. Introduction

Periodontitis is characterized by a pathological process triggered by the host response against pathogenic bacteria present in the dental biofilm. It is a chronic inflammatory disease that affects the periodontium resulting in tissue destruction and even in the loss of the dental organ [1]. Host immune response against this infection leads to the production of inflammatory mediators. Cytokines such as interleukin-1*β* and tumor necrosis factor-*α* are the primary mediators responsible for stimulating the production of secondary mediators like chemokines and cyclooxygenases (COX) [2]. These inflammatory molecules activate osteoclasts and induce bone resorption as a result of an exacerbated host response. Thus, understanding the regulation of proinflammatory mediators and their effects in periodontal tissues has been the objective of many studies [2–6].

Besides pathological conditions, orthodontic tooth movement also stimulates those biological mediators in response to therapeutic mechanical forces. Tooth displacement occurs as a result of periodontal tissues remodeling process, which is predominantly characterized by bone resorption and bone formation on pressure and tension sides, respectively. PDL cells are constantly subjected to several types of mechanical forces, such as compression, tension, and shear stress. In addition, they are considered as mechanoresponsive cells that mediate the response of the connective tissue to mechanical loading [7]. During orthodontic movement, several proinflammatory mediators are synthesized and released, especially cytokines and prostaglandins, playing an important role in bone remodeling. However, these molecules may also interfere with the underlying disease bacterial-induced inflammatory process and exacerbate periodontitis [8]. Although some studies [8–10] have been conducted to understand the effects of concomitant periodontal disease and orthodontic movement on periodontal tissues, the interactions between periodontitis and biomechanical loading are as yet not well established.

Among the many molecules involved in the inflammatory process, COX is highly expressed during both periodontal disease and orthodontic tooth movement. After several types of stimuli, the membrane phospholipids of some cells release arachidonic acid, which is catalyzed by COX into prostanoids like prostaglandins and thromboxane. Two isoforms of COX are described, COX1 and COX2. COX1 is constitutively expressed in many tissues and is required to maintain organ and tissue homeostasis. In contrast, COX2 expression is induced by proinflammatory cytokines and lipopolysaccharide. COX2 is responsible for prostaglandin E2 (PGE2) production [11], which has an important role in the pathogenesis of periodontal diseases. High levels of PGE2 are detected in the gingiva and gingival crevicular fluid of patients with periodontal disease [12–15], acting as inflammatory mediator. Also, PGE2 has been associated with bone resorption during the progression of periodontal diseases [14, 16] by stimulating and activating osteoclast production [17] and by upregulating receptor activator of nuclear factor kappa-B (RANK) ligand (RANKL) expression [18]. Moreover, blocking endogenous PGE2 production with indomethacin has been shown to significantly inhibit the increase of osteoclasts by LPS-induced COX2 [19]. In addition to the role in the disease process, some studies have demonstrated high expression of COX2 and/or PGE2 in periodontal ligament (PDL) cells after* in vitro* mechanical loading [20–25] and high PGE2 level in gingival crevicular fluid during orthodontic movement at both compression and tension sides [26], suggesting its participation in bone remodeling process [7]. This role of PGE2 in bone remodeling can be confirmed due to the increase in orthodontic tooth movement achieved after PGE2 administration [27–30]. Furthermore, COX2/PGE2 were demonstrated to be responsible for RANKL upregulation in PDL cells under mechanical stress* in vitro* [21].

RANKL is a key molecule in osteoclast differentiation and activation. Increased level of this molecule is detected in periodontal disease and orthodontic tooth movement [21, 31]. RANKL effects are counteracted by osteoprotegerin (OPG) and the balance between them regulates bone resorption [32]. The level of OPG in periodontitis seems to be lower when compared to that in healthy patients [31], but OPG regulation during mechanical stress is still uncertain [33, 34]. Some reports suggest that OPG expression remains unchanged while other reports suggest that its expression is upregulated in PDL cells subjected to biomechanical loading [21, 34, 35]. The fact is that RANKL and OPG are involved in PDL and bone remodeling.

However, no previous study has evaluated the expression of those important molecules COX2/PGE2 and RANKL/OPG when both conditions, bacterial challenge and mechanical force, are concomitantly present. Thus, the aim of the present study was to evaluate* in vitro* whether biomechanical loading modulates bacterial regulation of proinflammatory and bone remodeling mediators in PDL cells.

## 2. Materials and Methods

### 2.1. Cell Culture

The experiment was approved by the Ethics Committee of the University of Bonn and informed parental consent was obtained. Human periodontal ligament (hPDL) fibroblasts were used. Cells were obtained from six periodontally healthy donors, who underwent tooth extraction for orthodontic reasons. Cells were derived from the middle third of the tooth roots and maintained in DMEM (Dulbecco's Modified Eagle's Medium, Invitrogen, Karlsruhe, Germany) supplemented with 10% FBS (fetal bovine serum, Invitrogen), 100 units of penicillin, and 100 *μ*g/mL of streptomycin (Biochrom, Berlin, Germany) at 37°C in a humidified atmosphere of 5% CO_2_. Cells were seeded between passages 3–5 (1 × 10^5^ cells/well) on 6-well BioFlex collagen-coated culture plates (Flexcell International, Hillsborough, NC, USA) and grown to 80% confluence. FBS concentration was reduced to 1% 24 hours prior to experiments in order to avoid interference from its components and to synchronize the cell cycle.

### 2.2. Cell Stimulation

Cells were stimulated with the inactivated oral pathogen* Fusobacterium nucleatum* ATCC 25586 (OD_660 nm_: 0.025, 0.05, and 0.1) in order to mimic cell-microbial interactions* in vitro*. In an anaerobic atmosphere, bacteria strain was precultivated for 48 hours on Schaedler Agar plates (Oxoid, Basingstoke, United Kingdom). Subsequently, bacteria were suspended in PBS (OD_660 nm_ = 1, equivalent to 1.2 × 10^9^ bacterial cells/mL) and subjected twice to ultrasonication (160 W for 15 min). Different OD concentrations were used in the first experiment to evaluate the dose response of PDL cells stimulated with* F. nucleatum* ATCC 25586. Afterwards, the lowest concentration capable of upregulating COX2 was chosen (OD 0.025) and used in the subsequent experiments. As in previous experiments, a strain device (CESTRA) developed at the University of Bonn was used to apply biomechanical forces to cells [36–38]. In addition to bacterial challenge, biomechanical forces were simulated by the application of cyclic tensile strain (CTS) of low (CTSL, 3%) and high (CTSH, 20%) magnitudes at a rate of 0.05 Hz. PDL fibroblasts were exposed to* F. nucleatum* ATCC 25586, to CTS, and to their combinations for 1 day and 3 days. Moreover, cells were stimulated with* F. nucleatum* ATCC 25586 in the presence and absence of 10 *μ*g/mL blocking antibodies against TLR2 (mouse anti-human TLR2 mAb TL2.1, 16-9917-82, eBioscience, San Diego, CA, USA) and TLR4 (mouse anti-human TLR4 HTA125, 16-9917-82, eBioscience) for 1 day. Cell viability of treated and control cells was >95%.

### 2.3. Quantitative RT-PCR

Total RNA extraction was performed using RNeasy Mini Kit (Qiagen, Hilden, Germany) according to manufacturer's protocol. RNA concentration was determined by NanoDrop ND-1000 (Thermo Fisher Scientific, Wilmington, DE, USA) spectrophotometer. 500 ng of total RNA was reversely transcribed using the iScript Select cDNA Synthesis Kit (Bio-Rad, Munich, Germany) at 42°C for 90 min followed by 85°C for 5 min, following manufacturer's instructions. Using the iCycler iQ detection system (Bio-Rad), SYBR Green (Qiagen), and specific primers (QuantiTect Primer Assay, Qiagen), gene expression of glyceraldehyde-3-phosphate dehydrogenase (GAPDH), COX2, OPG, and RANKL was evaluated by quantitative RT-PCR. One microliter of cDNA was amplified as a template in a 25 *μ*L reaction mixture containing 12.5 *μ*L of 2x QuantiFast SYBR Green PCR Master Mix (Qiagen), 2.5 *μ*L of primers, and RNase free water. The PCR mixture was heated initially at 95°C for 5 min and then followed by 50 cycles of denaturation at 95°C for 10 s and combined annealing/extension at 60°C for 30 s. This analysis was performed in triplicate. Data were analyzed using the comparative threshold cycle method.

### 2.4. ELISA

Commercially available ELISA kits (DYC4198-2, DY805, DY626, R&D Systems Europe, Abingdon, United Kingdom, and HZ-5203, Hölzel Diagnostika, Cologne, Germany) were used for ELISA analyses according to the manufacturer's instructions to measure the levels of COX2 in cell lysates and of OPG, soluble RANKL, and PGE2 in cell supernatants. Using a microtiter plate reader (POWERWAVE X; BioTek Instruments, Winooski, VT, USA) the absorbance was determined at 450 nm with wavelength correction at 540 nm. COX2 data were normalized by total protein measured using Pierce BCA Protein Assay Kit (23227, Thermo Scientific, Pierce Biotechnology, Rockford, USA), while OPG, RANKL, and PGE2 data were normalized by the numbers of cells in the wells.

### 2.5. Statistical Analysis

Statistical analysis of the data was performed with GraphPad Prism 5 software (GraphPad Software Inc., San Diego, USA) using mean ± standard deviation. One-way analysis of variance test (ANOVA) followed by Dunnett's or Tukey's post hoc tests was used to determine the presence of significant differences among experimental groups. Significant differences were considered when *P* < 0.05. All experiments were performed in triplicate and repeated at least twice.

## 3. Results

### 3.1. Stimulation of COX2 and OPG Expressions by* F. nucleatum* ATCC 25586

**Figure 1:**
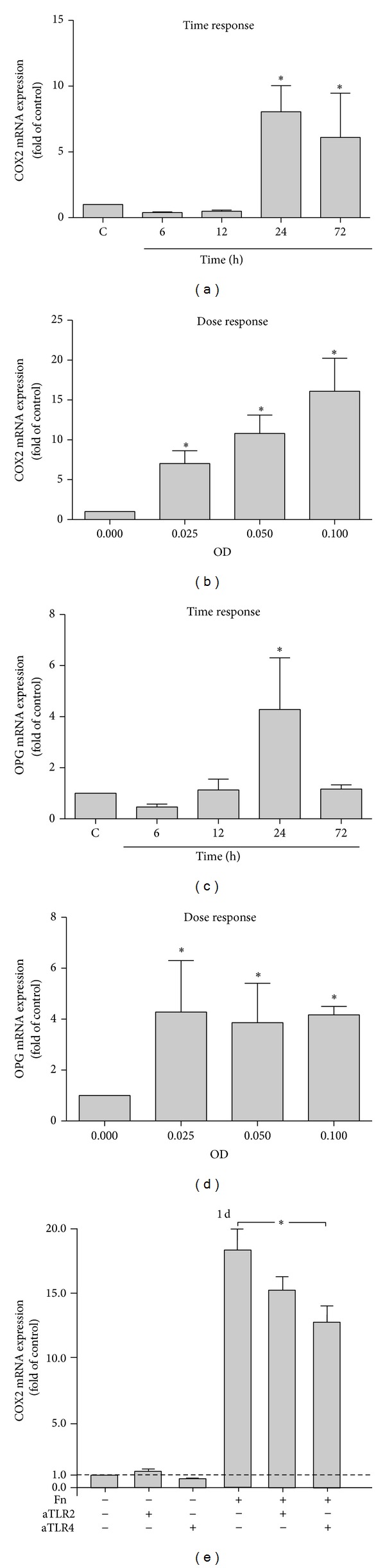
(a) COX2 expression in PDL cells stimulated by* F. nucleatum* ATCC 25586 over time. (b) COX2 expression in PDL cells stimulated by various concentrations of* F. nucleatum* ATCC 25586 at 1 day. (c) OPG expression in PDL cells stimulated by* F. nucleatum* ATCC 25586 over time. (d) OPG expression in PDL cells stimulated by various concentrations of* F. nucleatum* ATCC 25586 at 1 day. (e) COX2 expression in PDL cells stimulated by* F. nucleatum* ATCC 25586 (OD 0.025) in the presence or in the absence of anti-TLR2 or anti-TLR4 antibodies at 1 day. *Significant difference between groups (*P* < 0.05).

COX2 and OPG were constitutively produced by PDL cells. To mimic an inflammatory environment, cells were stimulated with* F. nucleatum* ATCC 25586 leading to a significant (*P* < 0.05) upregulation of the COX2 and OPG mRNA expression in a time-dependent manner. As shown in Figures 1(a) and 1(c),* F. nucleatum* ATCC 25586 had no significant effect on COX2 and OPG mRNA expression up to 12 hours, but at 24 hours it significantly (*P* < 0.05) increased the COX2 and OPG expressions.* F. nucleatum* ATCC 25586 enhanced significantly (*P* < 0.05) the mRNA expression of COX2 in a dose-dependent manner, whereas OPG was not influenced by the varying concentrations of bacteria at 24 hours (Figures 1(b) and 1(d)).

### 3.2. Stimulation of COX2 Expression by* F. nucleatum* ATCC 25586 via TLRs

In order to analyze whether* F. nucleatum* ATCC 25586 uses TLRs to upregulate COX2, cells were stimulated with* F. nucleatum* ATCC 25586 in the presence and absence of blocking antibodies against TLR2 and TLR4 for 24 hours. The results demonstrated that COX2 mRNA expression was decreased after treating PDL cells with anti-TLRs (Figure 1(e)). The inhibition of the* F. nucleatum*-induced COX2 expression occurred for both anti-TLRs being more evident and significant (*P* < 0.05) for anti-TLR4.

### 3.3. Regulation of* F. nucleatum-*Stimulated COX2 and PGE2 Syntheses by CTS

**Figure 2:**
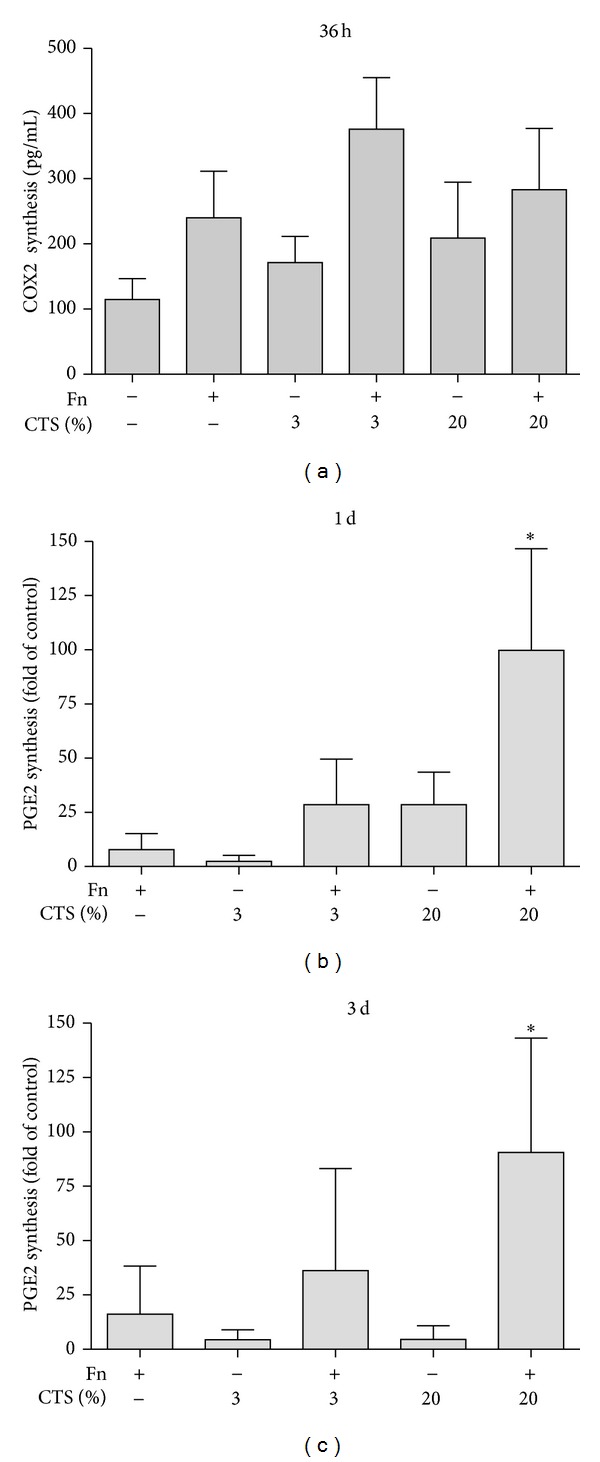
(a) Synthesis of COX2 in lysates of PDL cells treated with* F. nucleatum* ATCC 25586 and/or cyclic tensile strain (CTS) of low (CTSL, 3%) and high (CTSH, 20%) magnitudes at 36 hours. (b and c) Production of PGE2 in supernatants of PDL cells treated with* F. nucleatum* ATCC 25586 and/or CTSL and CTSH at 1 day (b) and 3 days (c). *Significant difference compared to other groups (*P* < 0.05).

In order to study whether biomechanical loading modulates the* F. nucleatum*-induced effects on COX2 and PGE2, CTS was applied to PDL fibroblasts. As evidenced by ELISA, CTSL and CTSH alone had only small effects on COX2 and PGE2 syntheses. However, biomechanical loading modulated the effects of* F. nucleatum* ATCC 25586 on COX2 and PGE2 in PDL cells. When PDL cells were concomitantly stimulated with CTS and* F. nucleatum* ATCC 25586, CTS tended to increase the* F. nucleatum*-induced effects on COX2 production (Figure 2(a)). Furthermore, CTS aggravated the* F. nucleatum*-induced effects on the PGE2 production (Figures 2(b) and 2(c)). As compared to CTSL, CTSH caused a more pronounced and significant increase in the* F. nucleatum*-induced PGE production (*P* < 0.05) at 1 day and 3 days (Figures 2(b) and 2(c)).

### 3.4. Regulation of* F. nucleatum-*Induced RANKL and OPG Expressions by CTS

**Figure 3:**
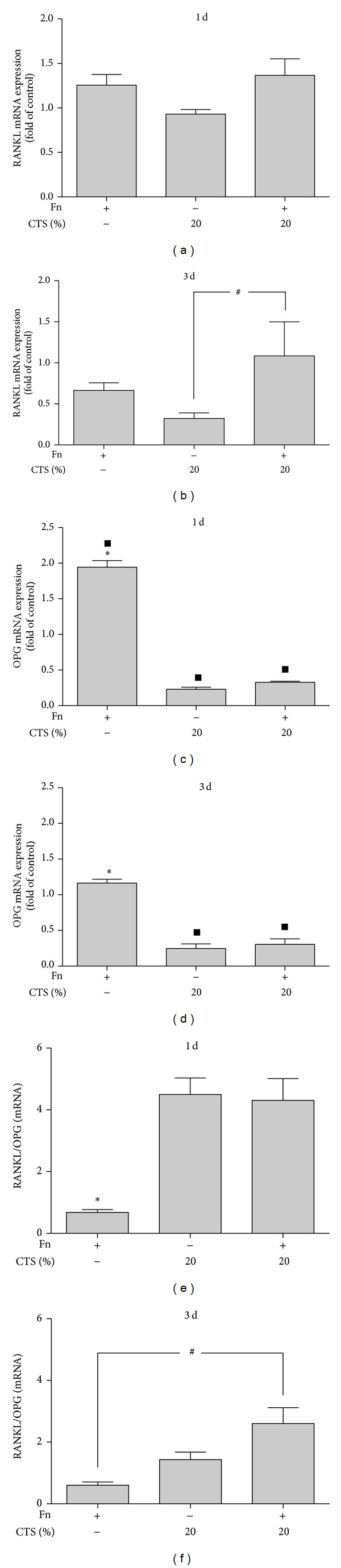
(a and b) RANKL expression in PDL cells stimulated by* F. nucleatum* ATCC 25586 and/or CTSH at 1 day (a) and 3 days (b). (c and d) OPG expression in PDL cells stimulated by* F. nucleatum* ATCC 25586 and/or CTSH at 1 day (c) and 3 days (d). (e and f) RANKL/OPG mRNA ratio in PDL cells stimulated by* F. nucleatum* ATCC 25586 and/or CTSH at 1 day (e) and 3 days (f). *Significant difference compared to other groups (*P* < 0.05), ^▪^significant difference compared to control (*P* < 0.05), and ^#^significant difference (*P* < 0.05).

**Figure 4:**
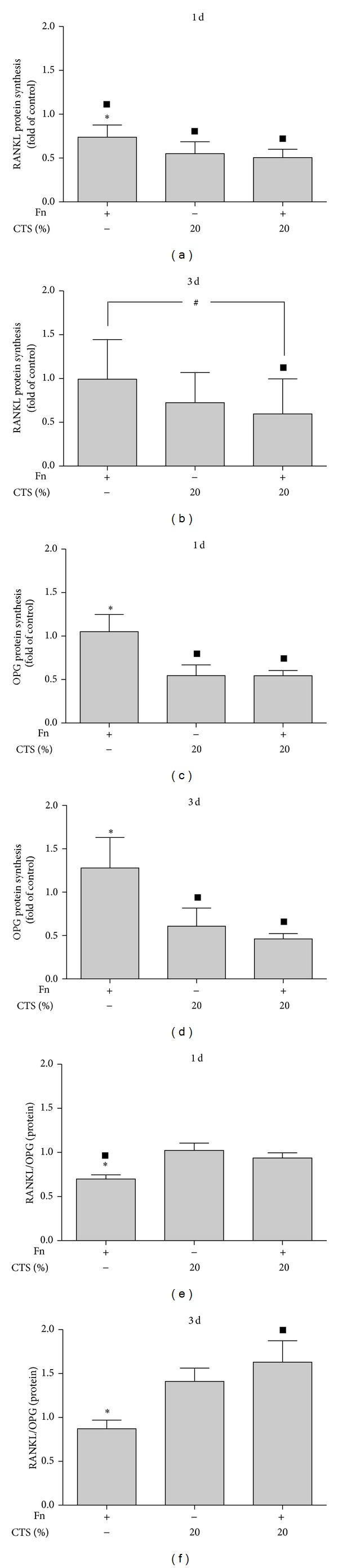
(a and b) Synthesis of RANKL protein in supernatants of PDL cells stimulated by* F. nucleatum* ATCC 25586 and/or CTSH at 1 day (a) and 3 days (b). (c and d) Synthesis of OPG protein in supernatants of PDL cells stimulated by* F. nucleatum* ATCC 25586 and/or CTSH at 1 day (c) and 3 days (d). (e and f) RANKL/OPG protein ratio in supernatants of PDL cells stimulated by* F. nucleatum* ATCC 25586 and/or CTSH at 1 day (e) and 3 days (f). *Significant difference compared to other groups (*P* < 0.05), ^▪^significant difference compared to control (*P* < 0.05), and ^#^significant difference (*P* < 0.05).

Next, we investigated the expression of RANKL and OPG in PDL cells challenged with* F. nucleatum* ATCC 25586 in the presence and absence of biomechanical loading.* F. nucleatum* ATCC 25586 tended to increase the RANKL expression at 1 day but not at 3 days, and significantly (*P* < 0.05), despite discrete, upregulated the OPG expression after both 1 day and 3 days (Figures 3(a)–3(d)). Whereas CTSL had no effects on RANKL and OPG expressions (data not shown), CTSH caused a significant (*P* < 0.05) upregulation of the* F. nucleatum*-induced RANKL expression at 3 days and a significant (*P* < 0.05) downregulation of the* F. nucleatum*-induced OPG expression at 1 day and 3 days (Figures 3(a)–3(d)). Furthermore, CTSH increased significantly the RANKL/OPG ratio in the presence and absence of* F. nucleatum* ATCC 25586 at both time points (Figures 3(e) and 3(f)). As shown in Figures 4(a)–4(f), the effects of biomechanical strain were also observed at protein level, as analyzed by ELISA.

## 4. Discussion

Many attempts have been made to study the effects of inflammatory and/or mechanical stimulation on PDL cells [37–40]. Occasionally the results are ambiguous especially due to differences in methodology such as culture conditions, type of inflammatory induction, type of mechanical apparatus, and type of strain regime. Our study aimed to investigate* in vitro* whether biomechanical loading would modulate bacterial regulation of important proinflammatory and bone remodeling mediators in PDL cells. The main finding of this study was that a CTS stimulus enhanced the* F. nucleatum*-induced increase of COX2 and PGE2. The association of CTSH and* F. nucleatum* ATCC 25586 resulted in an RANKL/OPG ratio that is significantly higher when compared to* F. nucleatum* ATCC 25586 alone.

*F. nucleatum* ATCC 25586 upregulates COX2 via TLRs signaling in PDL cells, especially by TLR4 due to the fact that preincubation with TLR4 antibody resulted in significant inhibition of the* F. nucleatum*-induced COX2 stimulation. Also,* F. nucleatum* is probably using other pathways for signaling in PDL cells as our data from the TLR inhibition experiment showed only partial inhibition of COX2 expression after blocking the TLRs 2 and 4. In addition,* F. nucleatum* was previously shown to stimulate TLR2 and 4 expressions in PDL cells [41]. Mechanical force driven by CTS to PDL cells increased COX2 production as previously demonstrated [11, 37]. When PDL cells were subjected to both stimuli, bacterial and mechanical force, COX2 production showed a tendency to be higher than the effect of each stimulus alone, and PGE2 expression and production were significantly increased in this situation. Thus, mechanical force increased the effect of* F. nucleatum* ATCC 25586 on COX2-PGE2 production exhibiting a proinflammatory effect.

Studies have reported that PGE2 mediates bone resorption through the activation of osteoclasts and RANKL in response to mechanical stress* in vitro* [11] and* in vivo* [25]. Due to this fact, we have decided to investigate whether RANKL and OPG expression and production would be modulated by* F. nucleatum* ATCC 25586 and CTS in PDL cells.* F. nucleatum* ATCC 25586 induced the expression and synthesis of RANKL and OPG in PDL cells at both time points, 1 day and 3 days. CTSH in the presence of* F. nucleatum* ATCC 25586 inhibited the expression and synthesis of OPG while it stimulated the increase in RANKL. As a result, the RANKL/OPG ratio was low for the groups stimulated by* F. nucleatum* ATCC 25586 alone and high for the other two groups, CTSH alone and CTSH associated with* F. nucleatum* ATCC 25586.

In our study, CTS aggravated* F. nucleatum*-induced increase in the production of COX2 and PGE2 and reduced the expression and production of OPG, leading to an increase in RANKL/OPG ratio in this group compared to* F. nucleatum* ATCC 25586 alone. These results suggest an exacerbated proinflammatory and bone resorptive effect of CTS in the presence of bacteria. Our results corroborate in part other previous studies in that biomechanical loading can exert proinflammatory effects under inflammatory conditions in PDL cells [37, 40, 42]. All of these studies applied CTS of low and high strains in cells treated with IL-1*β*. Some of them reported that high strain was proinflammatory and enhanced the IL-1*β*-induced production of inflammatory mediators [40, 42], while another study reported a proinflammatory effect for both strains only at 1 day [37]. Long et al. [40] have detected an anti-inflammatory effect of CTSL and Nokhbehsaim et al. [37] found this same effect when cells were subjected to long-term application of CTS. In addition to the proinflammatory effect of biomechanical loading, mechanical stress driven by hydrostatic pressure has been shown to intensify the production of proinflammatory cytokines in PDL cells stimulated with periodontopathogenic bacteria [39]. The contradiction in the results exists especially due to differences in experimental features, for example, the type of inflammatory mediator that is being evaluated and the type of force that is being used.

Some studies reported that PDL cells express RANKL in response to mechanical stress [21, 35], while other studies reported PDL cells do not express RANKL [33] or express low levels of RANKL [34]. Regarding the expression of OPG, downregulation was detected in response to CTS in PDL cells [33]. Corroborating this study, our results also revealed downregulation of OPG in response to CTS and, in addition, an inhibitory effect of CTS on* F. nucleatum*-induced increase in OPG expression and protein synthesis. On the other hand, in another study, PDL cells subjected to CTS have expressed high levels of OPG and this effect was inhibited after concomitant stimulation with LPS [34]. Although contradictions can be observed in the literature regarding OPG expression after CTS stimulus in all studies, RANKL/OPG ratio increased when both CTS and inflammatory challenge were associated, suggesting that those conditions together have a bone resorptive effect.

In addition, when osteoblasts are cultured with inflammatory conditioned medium and subjected to CTS, an upregulation of c-fos and reduction of osteogenicity were observed [43]. C-fos is a transcription factor important for the activation of genes involved in osteoclastogenesis [44]. This study shows that CTS in combination with inflammation is impairing the osteogenic capacity of osteoblasts. Although we have not evaluated c-fos, our* in vitro* study demonstrates a proinflammatory and bone resorptive (increase in the RANKL/OPG ratio) effect of CTS when associated with* F. nucleatum* ATCC 25586.

Periodontitis has a polymicrobial nature as it is originated from a complex bacterial biofilm. In order to mimic an inflammatory infection* in vitro*,* F. nucleatum* ATCC 25586, which is a gram-negative and anaerobic microorganism associated with both gingivitis and periodontitis, was used. In our study,* F. nucleatum* ATCC 25586 upregulated COX2, PGE2, and OPG syntheses in PDL cells.* F. nucleatum* ATCC 25586 is considered as a bridge bacterium because it is located in the middle of the subgingival biofilm inducing the adhesion of late colonizers during plaque development by coaggregation [45, 46].* F. nucleatum* ATCC 25586 can invade some cells and help other periodontopathogens to invade host cells [47, 48]. However, more studies are necessary to evaluate whether other microorganisms associated with periodontal diseases are also able to activate the production of the mediators that we have evaluated in the present study. Another limitation of this study is that it did not examine the involvement of COX2-PGE2 on the RANKL/OPG ratio when cells were concomitantly stimulated by biomechanical loading and bacteria. This could be shown by blocking COX2 with indomethacin, for example. However, a recent study demonstrated that COX2 inhibition with celecoxib resulted in RANKL downregulation and osteoclastogenesis reduction in PDL cells stimulated with heat-inactivated bacteria [49].

The sRANKL ELISA kit used in the present study can be interfered by OPG, according to the manufacturer's datasheet. In our samples, the OPG concentration was higher than 156 pg/mL, so that interference cannot be completely excluded in our experiments. However, this ELISA kit is frequently used by other investigators and it is difficult to avoid OPG in the samples.

The periodontium is constantly subjected to complex biomechanical forces such as mastication, orthodontic tooth movement, and functional occlusal habits. In the present study, we investigated the interactions of biomechanical forces and inflammatory signals in PDL cells. Our results revealed that biomechanical loading enhanced the* F. nucleatum*-induced upregulation of COX2 and PGE2 production and the RANKL/OPG ratio in PDL cells. These findings indicate that biomechanical loading can aggravate the destructive effects of inflammation on periodontal tissues during periodontitis. The strain regimens used in our experiments showed that biomechanical loading has a proinflammatory effect favoring the actions of* F. nucleatum* ATCC 25586. Previous studies corroborate our results [37, 40, 42].

In summary, our findings provide original evidence that CTS can enhance the synthesis of molecules associated with inflammation and bone resorption. Therefore, biomechanical, such as orthodontic or occlusal, loading may enhance the bacterial-induced inflammation and destruction in periodontitis.
